# Effective CpG Delivery Using Zwitterion-Functionalized Dendrimer-Entrapped Gold Nanoparticles to Promote T Cell-Mediated Immunotherapy of Cancer Cells

**DOI:** 10.3390/bios12020071

**Published:** 2022-01-27

**Authors:** Huan Chen, Yiming Zhang, Lulu Li, Rui Guo, Xiangyang Shi, Xueyan Cao

**Affiliations:** State Key Laboratory for Modification of Chemical Fibers and Polymer Materials, Shanghai Engineering Research Center of Nano-Biomaterials and Regenerative Medicine, College of Chemistry, Chemical Engineering and Biotechnology, Donghua University, Shanghai 201620, China; chenhuan800@gmail.com (H.C.); 2200737@mail.dhu.edu.cn (Y.Z.); 2200744@mail.dhu.edu.cn (L.L.); ruiguo@dhu.edu.cn (R.G.)

**Keywords:** dendrimers, gold nanoparticles, CpG-ODN, immunotherapy, T cells

## Abstract

Recently, cell-based immunotherapy has become one of the most promising ways to completely eliminate cancer. The major challenge is to effectively promote a proper immune response to kill the cancer cells by activated T cells. This study investigated the effect of T cell-mediated immunotherapy trigged by Au DENPs-MPC (zwitterion 2-methacryloyloxyethyl phosphorylcholine (MPC)-functionalized dendrimer-entrapped gold nanoparticles) loading oli-godeoxynucleotides (ODN) of unmethylated cytosine guanine dinucleotide (CPG). Here, we first synthesized Au DENPs-MPC, evaluated their capability to compress and transfect CpG-ODN to bone marrow dendritic cells (BMDCs), and investigated the potential to use T cells stimulated by matured BMDCs to inhibit the growth of tumor cells. The developed Au DENPs-MPC could apparently reduce the toxicity of Au DENPs, and enhanced transfer CpG-ODN to the BMDCs for the maturation as demonstrated by the 44.41–48.53% increase in different surface maturation markers. The transwell experiments certificated that ex vivo activated T cells display excellent anti-tumor ability, which could effectively inhibit the growth of tumor cells. These results suggest that Au DENPs-MPC can deliver CpG-ODN efficiently to enhance the antigen presentation ability of BMDCs to activate T cells, indicating that T cells-based immunotherapy mediated by Au DENPs-MPC loaded with CpG-ODN may become the most promising treatment of cancer.

## 1. Introduction

In the last few decades, immunotherapy has become an important and promising treatment for cancer [1,2], which can stimulate or boost our immune system to defend cancer cells in a much more robust and smarter manner [3]. The immune system detects abnormal cells and prevents the growth of many cancers with the help of tumor-infiltrating lymphocytes (TILs). However, cancer cells have ways to avoid destruction by the immune system. Immunotherapy can strengthen multiple antitumor capabilities to kill cancer cells in-site and achieve anti-metastasis and anti-relapse effects by strengthening the response of the immune system [4]. Recently, cell-based immunotherapy has been believed to be one of the most effective clinical therapy modalities for tumor therapy [5,6]. The patient’s immune system can eliminate tumor cells mostly through cytotoxic T lymphocyte (CTL) mediation [7,8]. While some of the stimulators are unable to reach high levels of immune response owing to the phenomenon of immune escape [9], it is necessary to find a much more efficient stimulator for immune cells.

Dendritic cells (DCs) are the unique and mighty antigen presenting cells (APCs), where only APCs could stimulate primary T lymphocytes and trigger the immunologic responses of cytotoxic T lymphocytes [10]. The immature DCs (iDCs) will become mature DCs (mDCs) in vitro with the procedure of stimulation by some stimulators, and then will stimulate T cells, especially CD8+ killer T cells and CD4+ helper T cells, to boost different antitumor immune reactions [11,12]. The immune stimulating DNA containing unmethylated cytosine-guanine (CpG) motifs have been successfully used as adjuvants to increase immune responses [13,14]. By MyD88-dependent nuclear factor-KB (NF-κB) and mitogen-activated protein kinase (MAPK) signaling pathways [15], the Toll-like receptor 9 (TLR9) on the surfaces of DCs can directly activate iDCs to mDCs when it detects CpG-ODN.

However, owing to various systemic and intracellular obstacles in gene therapy, containing fast degradation, poor cellular uptake, and inefficient endosomal escape, some special transport ways may be needed to effectively deliver genes to the cell coleus or cytosol [16,17]. Therefore, it is a challenge to find an outstanding gene carrier that could efficiently transport the exogenous nucleic acids into cells. Nonviral delivery systems have attracted extensive attention thanks to their properties of easy preparation and modification, high safety and genetic loading capacity, and perfect delivery efficiency [18,19]. Fifth-generation (G5) polyamidoamine (PAMAM) dendrimers are a kind of highly branched and symmetrical polymer with a precise molecular structure and abundant surface functional groups, which make it useful for gene delivery [20,21]. Modifications for dendrimers are necessary to overcome a series of shortcomings (e.g., high cytotoxicity and low gene transfection efficiency) [22,23]. Metallic and metal oxide NPs have been widely used in cell-based immunotherapy and other biomedical fields in recently years [24,25,26]. In our previous study, dendrimer-entrapped Au NPs (Au DENPs) showed more high gene delivery efficiency and lower cytotoxicity than it alone [27,28]. This is because of the truth that entrapped Au NPs can help to maintain the 3-dimensional spherical morphology of dendrimers, allowing more efficient compaction of DNA [29,30]. Meanwhile, the cytotoxicity of the gene vector was also reduced after dendrimer entrapment of Au NPs. After surface modification, it can further decrease the cytotoxicity and strengthen the efficiency of gene delivery upon Au DENPs [31,32].

To reduce the nonspecific adsorption and improve the transport capacity to immune cells, the antifouling effect of the delivery vectors is also worthy of attention. Zwitterionic materials have attracted more attention in the field of biomaterials than other materials owing to a range of advantages (e.g., ultralow nonspecific protein adsorption, bacterial adhesion, and biofilm formation) in recent years [33]. Zwitterions can form stronger hydration shells through ion–dipole interaction with denser and tighter adsorbed water [34], making them preferable substitutes for other materials, such as PEG [35]. To enhance the efficiency of gene delivery, it is needed to remove the existence of serum protein in the medium. This is because of the strong interaction between serum protein and positively charged vector/gene complexes, leading to low efficiency of cellular uptake and gene delivery [36]. However, it is very conflicting that the cells will become weak owing to lack of adequate nutritional in the serum-free medium, which also affect the gene delivery efficiency. Hence, it would be significant to discover a new protein-resistant carrier program to retain the formed positively charged vector/gene polyplexes to be intact in a serum culture environment for highly efficient gene delivery. Liu et al. and Xong et al. modified G5.NH_2_ with the zwitterion: MPC and carboxy betaine acrylamide (CBAA) to bear the G5.NH_2_ with outstanding compatibility with protein and cells [37,38].

**Scheme 1:**
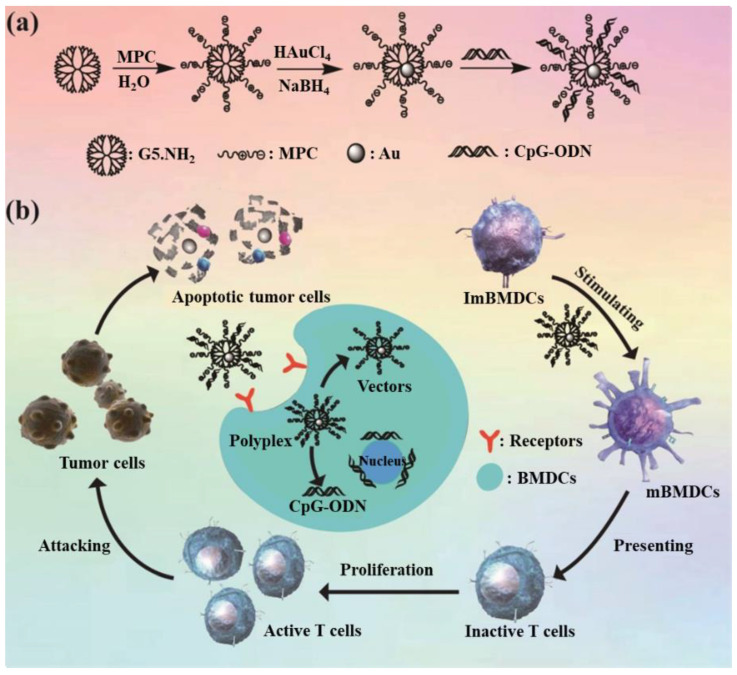
Synthesis of Au DENPs-MPC (**a**) and the anti-tumor immunotherapy route (**b**).

In our study, we explored a novel T cell-based tumor immunotherapy induced by CpG-loaded zwitterion-functionalized Au DENPs. G5.NH_2_ was used as a template to synthesize Au DENPs-MPC (Au DENPs-MPC) (Scheme 1). The cytocompatibility of Au DENPs-MPC on BMDCs was assessed by the MTT kit. Au DENPs-MPC were completely characterized via different techniques. Then, the surface antigens on BMDCs were detected by flow cytometry to certificate that they were successfully stimulated to maturation. The activation of T cells was detected by allogeneic mixed lymphocyte reaction (MLR) setting. The in vitro anti-tumor effect was detected by the transwell system and it indicated that T cells activated in vitro have a satisfactory anti-tumor effect. Therefore, Au DENPs-MPC can be served as an efficient gene delivery vector and DCs stimulator, and potentially enhance T cell-mediated tumor immunotherapy after loading with CpG-ODN. It also provides a good reference for the design of biosensors, and broadens the application field of biosensor.

## 2. Results and Discussion

### 2.1. Synthesis and Characterization of Au DENPs-MPC

**Figure 1:**
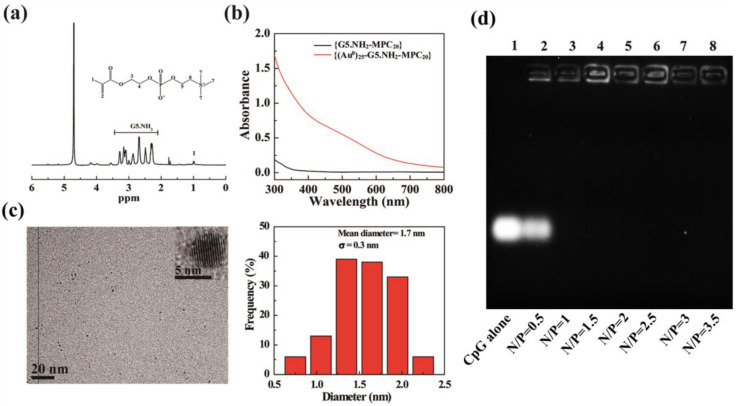
(**a**) ^1^H-NMR spectrum of Au DENPs-MPC. (**b**) UV/Vis absorption spectra of {G5.NH_2_-MPC_20_} and Au DENPs-MPC. (**c**) TEM image and size distribution histogram of Au DENPs-MPC. (**d**) Agarose gel electrophoresis of Au DENPs-MPC/CpG-ODN complexes at different N/P ratios (range from 0.5~3.5).

Based on the ^1^H-NMR (Figure 1a), there are about 21.2 MPCs attached to each G5.NH_2_ by integrating the areas of G5.NH_2_ proton peaks (range from 2.2 to 3.4 ppm) and MPC proton peaks (about −1.0 ppm). Figure 1b indicates the existence of Au NPs inside G5-NH_2_ due to the absorption peak at 520 nm. The Au NPs could also be visualized by transmission electron microscopy (TEM) (Figure 1c). It was shown that the Au NPs were spherical, and the average diameter was about 1.7 nm. In addition, the calculated Mws and mean numbers of primary amines of the G5.NH_2_ and Au DENPs-MPC vectors are shown in Table S1, respectively. The gel retardation results demonstrated that the Au DENPs-MPC showed the best ability to compress CpG-ODN, and the mobility of CpG-ODN could be retarded at N/P ratios of 1 or greater (Figure 1d).

Based on the above results, Au DENPs-MPC/CpG complexes with five different nitrogen phosphorus (N/P) ratios (1, 2, 4, 6, and 8) were selected to evaluate the hydrodynamic size and zeta potential. The average hydrodynamic sizes of the Au DENPs-MPC/CpG-ODN complexes were about 200 nm, and their average zeta potentials were about 15 mV, as shown in Table S2. Considering the optimal endocytosis efficiency and gene delivery efficiency [39,40], we chose the complexes with an N/P ratio of 2 for the subsequent studies.

### 2.2. In Vitro Cytotoxicity and Cellular Uptake Assays

**Figure 2:**
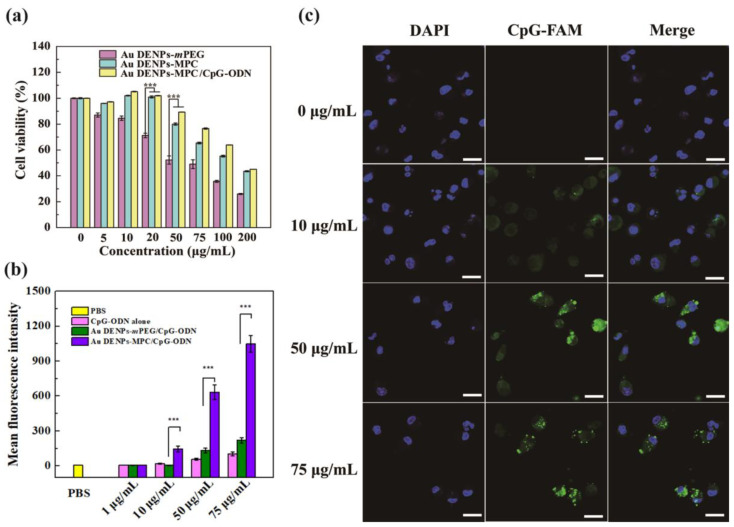
(**a**) MTT assay results of the BMDCs cultured with Au DENPs-mPEG, Au DENPs-MPC alone, and Au DENPs-MPC/CpG-ODN complexes at different concentrations for 24 h. (**b**) Flow cytometry results of BMDCs cultured with CpG-ODN alone, Au DENPs-mPEG/CpG-ODN, and Au DENPs-MPC/CpG-ODN complexes at different concentrations for 4 h. (**c**) Confocal fluorescence images results of BMDCs cultured with Au DENPs-MPC/CpG-ODN complexes at concentration of 50 µg/mL. The scale bar is 20 µm. *** *p* < 0.001.

Next, the cytocompatibility of the tested nanomaterials was evaluated, which was significant for the following experiments. According to the MTT assay results, Au DENPs-MPC showed lower cytotoxicity to BMDCs after being modified with MPC, indicating the outstanding cytocompatibility (Figure 2a). Meanwhile, the Au DENPs-MPC loaded with CpG-ODN could further decrease the cytotoxicity compared with Au DENPs-MPC alone, which is due to the decreased positive potential of the complexes after complexation of negatively charged CpG ODN [41], thereby improving the cytocompatibility of Au DENPs-MPC/CpG-ODN. It is noted that, when the concentration is 10 μg/mL, the cell viability is higher than 100%, which should be due to the lower cytotoxicity of the complexes under the lower concentrations. Even when the concentration of Au DENPs-MPC/CpG-ODN complexes was up to 50 μg/mL, the cell viability of BMDCs could still reach higher than 90%, indicating that the Au DENPs-MPC/CpG-ODN complexes were safe for subsequent experiments.

As shown in Figure 2b, the mean fluorescence intensity of CpG-ODN labeled with FAM (carboxyfluorescein) was increased obviously with the concentration of Au DENPs-MPC/CpG-ODN complexes and Au DENPs-*m*PEG/CpG-ODN complexes, respectively, while the intensity did not change obviously in the group of CpG-ODN alone (Figure 2b and Figure S1). This is because of the negative charge and easy degradation of CpG-ODN, which are not beneficial for uptake by cells. What is more, the decoration of zwitterion (MPC) onto the Au DENPs’ surface is able to efficiently degrade the adsorption of protein to Au DENPs-MPC/CpG-ODN complexes, thereby greatly reducing the influences of the presence of FBS [37]. Thus, Au DENPs-MPC/CpG-ODN showed about 4.7 times higher cellular uptake ability of BMDCs than Au DENPs-*m*PEG/CpG-ODN. In addition, the flow cytometry results were consistent with the results of confocal fluorescence detection (Figure 2c).

### 2.3. Maturation of BMDCs

**Figure 3:**
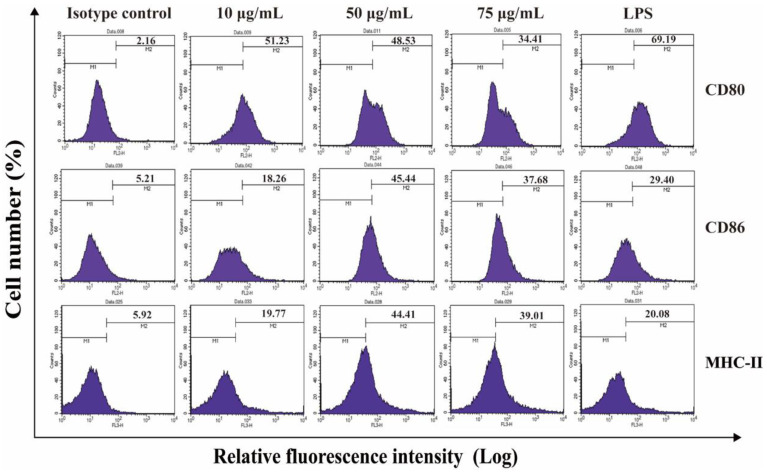
The expression detection results of different maturation markers were detected by flow cytometry and the isotype controls in panels are related to the determination background.

Maturation of BMDCs is the first step in immune response for the anti-tumor effect. Only mature BMDCs can present antigens and effectively activate T cells. In Figure S2, the expression of CD11c on the surfaces of BMDCs reached more than 75%, suggesting the high purity of BMDCs. The results from Figure S3 presented the extracted cell morphology, and were similar to those in the previous reports [42]. Compared with the group without any stimulation, the expression of BMDCs’ maturation markers, CD80, CD86 and MHC-II, were remarkably raised to 51.23%, 45.44%, and 44.41%, respectively, indicating the BMDCs were matured after incubated with 50 μg/mL Au DENPs-MPC/CpG-ODN (Figure 3). To further confirm the stimulation effect of the Au DENPs-MPC/CpG-ODN, liposome was also used as a positive control. In the liposome group, the expressions of CD86 and MHC-II were just 29.40% and 20.08%, respectively. This means that more CpG-ODN are transferred into cells and then stimulate the maturation of DCs by Au DENPs-MPC rather than liposome. It is notable that, although Au DENPs-MPC/CpG-ODN at 75 μg/mL showed much better cellular uptake efficiency, the expression levels of related cytokines are lower than those of Au DENPs-MPC/CpG-ODN at 50 μg/mL. This may be due to the increasing cytotoxicity of Au DENPs-MPC/CpG-ODN at high concentrations, which limits the function of CpG-ODN. According to the above results, the concentration of Au DENPs-MPC at 50 μg/mL was selected for the following tests.

### 2.4. T Cells’ Activation

**Figure 4:**
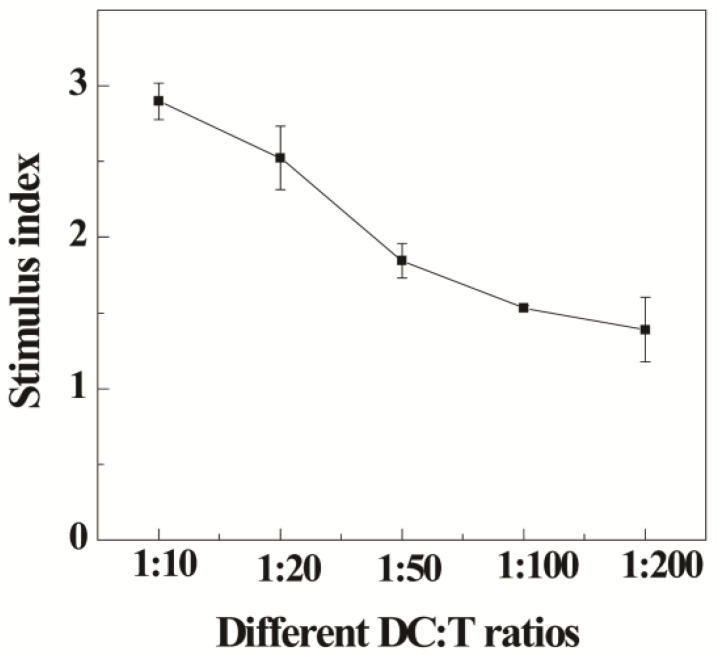
The proliferation result of T cells after co-culture with different ratio of mature BMDCs for 3 days by MTT assay.

Next, the activation of T cells in vitro through mature BMDCs (mBMDCs) was checked. Firstly, we checked the purity of the extracted T cells according to our previous reports [43]. The flow cytometry detection results (Figure S4) showed that the expression of CD3 reached above 90%, suggesting high purity of the extracted T cells [43]. Then, the proliferation of T cells was detected by the allogeneic MLR setting [44]. It is clear that BMDCs matured by the Au DENPs-MPC/CpG-ODN complexes could activate the proliferation of allogeneic T cells at a DC/T ratio higher than 1:200, and the highest stimulus index was 2.8 times higher than that of the control group (T cells without stimulation) (Figure 4).

Meanwhile, the flow cytometry results also showed that, after co-culturing T cells with mBMDCs, the expressions of CD4 and CD8 on the surface of T cells increased (Figure S5). Thus, Au DENPs-MPC/CpG-ODN complexes-treated BMDCs exhibited a remarkably enhanced capacity for presenting antigen to T cells for the stimulation of cell proliferation, showing that Au DENPs-MPC/CpG-ODN complexes were able to induce functional maturation of BMDCs.

### 2.5. In Vitro Anti-Tumor Effect of T Cells

**Figure 5:**
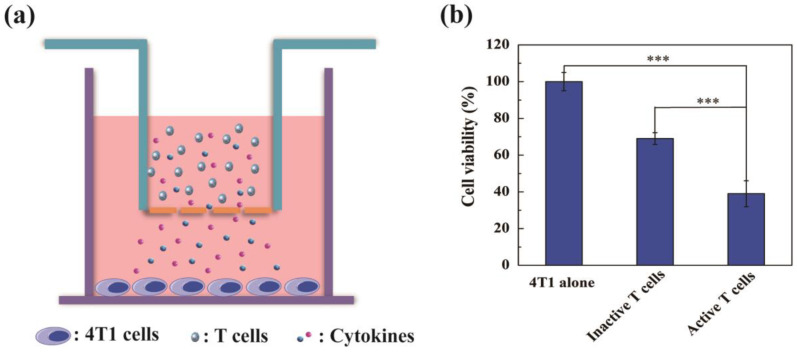
(**a**) Schematic illustration of T cells (upper) and tumor cell (lower) co-cultured using the transwell plates. (**b**) Cell viability of 4T1 after co-culture with different T cells. *** *p* < 0.001.

Considering the remarkable activation of T cells stimulated by Au DENPs-MPC/CpG-treated BMDCs, the in vitro antitumor effect of T cells was detected by the transwell system (Figure 5a). Based on our results, the activated T cells showed the most powerful and good inhibition effect of 4T1 cells, compared with inactivated T cells and 4T1 alone. When it was co-cultured with activated T cells for 24 h, the cell viability of 4T1 cells dropped to 37% (Figure 5b). It is believed that activated T cells in the upper chamber could secrete some abundant related cytokines, such as interferon-γ (IFN-γ) and tumor necrosis factor-α (TNF-α), to inhibit the growth of 4T1 cells in the lower chamber [45]. Meanwhile, the inactivated T cells group also showed a low inhibition effect on the growth of 4T1 cells, which may because the inactivated T cells also secrete a small amount of related cytokines to kill the 4T1 cells [45]. This showed that T cells activated by mBMDCs could be able to inhibit the growth of tumor cells by triggering the immune response.

## 3. Conclusions

In summary, we synthesized a kind of non-viral carrier Au DENPs-MPC with improved cytocompatibility and delivery efficiency of CpG-ODN for enhancing the antigen presentation ability of BMDCs to activate T cells. The results showed that Au DENPs-MPC could reduce the toxicity of Au DENPs and increase the efficiency of delivery of CpG-ODN to BMDCs. The expression of specific surface proteins detected by flow cytometry demonstrated the maturation of BMDCs and the activation of T cells. The transwell experiments certificated that ex vivo activated T cells display excellent anti-tumor ability, which could effectively inhibit the growth of tumor cells. These results show that the T cells-based immunotherapy mediated by Au DENPs-MPC loaded with CpG-ODN may become the most promising treatment of cancer.

## Data Availability

Data are contained within the article.

## Supplementary Materials

The following supporting information can be downloaded at https://www.mdpi.com/article/10.3390/bios12020071/s1, Table S1: Physicochemical parameters of the G_5_.NH_2_, {(Au^0^)_25_-G_5_.NH_2_-mPEG_20_}, and {(Au^0^)_25_-G_5_.NH_2_-MPC_20_} vectors. Table S2: Zeta potentials and hydrodynamic diameters of Au DENPs-MPC alone and the Au DENPs-MPC/CpG complexes at five different N/P ratios. Figure S1: Cellular uptake results of BMDCs detected by flow cytometry. Figure S2: The expression of CD11c on the surfaces of BMDCs related the purity of BMDCs was detected by flow cytometry. Figure S3: The inverted microscope images of mouse bone marrow-derived dendritic cells after induction for 1, 3, 5, and 7 days. Figure S4: The expression of CD3 on the surfaces of T cells related to the purity of T cells was detected by flow cytometry. Figure S5: The flow cytometry results of the expression of CD4 and CD8 on the surfaces of T cells. (a,b) T cells cultured for 3 days without stimulation. (c,d) T cells cultured with mBMDCs for 3 days. CD4 and CD8 were labeled with FITC (F1 channel).
