# Supplementary material for: Effective CpG Delivery Using Zwitterion-Functionalized Dendrimer-Entrapped Gold Nanoparticles to Promote T Cell-Mediated Immunotherapy of Cancer Cells
Source: Biosensors (Basel). 2022 Jan 27;12(2):71. doi: 10.3390/bios12020071 (PMC8869692; doi:10.3390/bios12020071)
Supplement: Supplementary file 1 [file biosensors-12-00071-s001.zip › biosensors-1556268-supplementary.pdf]

biosensors

Supplementary

Effective CpG Delivery Using Zwitterion-Functionalized Dendrimer-Entrapped Gold Nanoparticles to Promote T Cell-Mediated Immunotherapy of Cancer Cells

Materials

$G_5.NH_2$ PAMAM dendrimers were purchased from Dendritech, Inc. (Midland, United States). 2-methacryloyloxyethyl phosphorylcholine (MPC) was provided by Shanghai Yanyi Biotechnology Corporation (Shanghai, China). The Primary Amino Nitrogen (PANOPA) Assay Kit was obtained from Megazyme (Wicklow, Ireland). Agarose was from Gene Tech (Shanghai, China). Ethidium bromide (95%) and sodium borohydride ($NaBH_4$) were from Aldrich (St. Louis, MO). MTT and CCK-8 assay kit were acquired from Shanghai Sangon Biological Engineering Technology & Services Co., Ltd. (Shanghai, China). RPMI 1640, fetal bovine serum (FBS), penicillin, and streptomycin was from Lingfeng Chemical Reagent Co.,Ltd. (Shanghai, China). 4T1 cells were ob-tained from the Shanghai Institutes for Biological Sciences, the Chinese Academy of Sciences. Single-stranded CpG (5′-TCCATGACGTTCCTGACGTT-3′) was purchased from Sangon Biotech (Shanghai, China) Co., Ltd. All the antibodies and LPS were purchased from ebioscience (USA). Cytokines IL-4 and GM-CSF were purchased from BBI (USA). Nylon wool was purchased from Kisker, Germany. C57BL/6 mice of 6-8 weeks old were obtained from Shanghai Slake Animal Co., Ltd. (Shanghai, China).

Synthesis and Characterization of Au DENPs-MPC

The MPC was dissolved in H2O and the $G_5.NH_2$ was added into it. They were reacted for 3 days at the molar ratio of 20:1 to obtain the {$G5.NH_2$-MPC_{20}}. The $HAuCl_4$ and $NaBH_4$ were added into it reacted for 30 min to obtain the Au DENPs-MPC. The {$G5.NH_2$-MPC_{20}} was dissolved in D_2O and 1HNMR was characterized. Meanwhile, the {$G5.NH_2$-MPC_{20}} and Au DENPs-MPC were tested by UV/Vis spectrophotometry. The size and morphology of gold nanoparticles were observed by transmission electron microscopy (TEM, JEOL 2010F, Tokyo, Japan) at an accelerating voltage of 200 kV. The amount of primary amine was determined by a nitrogen assay kit (Table S1).

Gel electrophoresis Assay

The Au DENPs-MPC/CpG-ODN complexes were prepared at eight different N/P ratios of 0.125:1, 0.25:1, 0.5:1, 1:1, 2:1, 3:1, 4:1,5:1. CpG-ODN dosage per well was 1 μg. It was performed at voltage of 80 V for 30 min and the migration of CpG-ODN in the gel was analyzed by a FR-1000 gel image analysis system.

Hydrodynamic Size and Zeta Potential Detection

The five different N/P ratio (1:1, 2:1, 4:1, 6:1, 8:1) of the Au DENPs-MPC/CpG-ODN complexes were selected for the hydrodynamic size and zeta potential detection, and Au DENPs-MPC was used for control. DLS measurements of Au DENPs-MPC and Au DENPs-MPC/CpG-ODN were performed on a Zetasizer Nano ZS system (Malvern, UK) instrument with a standard 633 nm laser. The measurements were carried out at 25 °C and the intensity weighted approaches were used for the data analysis. All the sample measurements were acquired in triplicate and reported as an average and standard error.

Extraction and Culture of Mouse Bone Marrow-Derived Dendritic Cells

C57BL/6 mice were killed by cervical dislocation. The femoral and tibial epiphyses were clean off in PBS, and the bone marrow cavity was repeatedly washed by RPMI1640 medium until the bones became white. The Red Blood Cell Lysis Buffer was used to lyse red blood cells. RPMI 1640 medium was used to resuspend the cells and adjust its concentration to 1×10^6/mL. The extracted cells were seeded into 6-well plate, and at the same time, IL-4 and GM-CSF were added for induction at the concentration of 10 ng/mL and 20 ng/mL separately. The whole medium was changed at the first 2 days, then half of the medium was changed at the following 4 days. The immature dendritic cells were obtained at 7 day.

Detection of BMDCs Purity

To confirm the successful induction from bone marrow stem cells to BMDCs, the morphology of the BMDCs was observed at 1, 3, 5, and 7 days through the inverted microscope. If the morphology conforms to the properties, the cells would be collected at the 7th day for detection its purity. FITC-labled CD11c antibodies were used to incubate with the collected cells at 4°C in dark for 30 min. Excess unbound antibodies were washed away by PBS. Flow cytometry was used to detect the expression level of the antibody on the surface of the cells.

Flow cytometry was carried out using a Becton Dickinson FACS can analyzer equipped with a 15 mW, 488 nm, and air-cooled argon ion laser and a 40 mW and 635 nm HeNe laser. Data were collected based on the counting of 10,000 events. The assay was performed 3 times for each sample.

The Extract and Culture of T Cells

C57BL/6 mice were killed by cervical dislocation and immersed in 75% alcohol for 2-5 min. The spleen was obtained in sterile conditions and it was removed to PBS. The spleen was grinded in 400 mesh screen, and the lymphocytes in the spleen were separated using lymphocyte separation buffer. Then, the obtained lymphocytes were filtered by the nylon wool column for 6-8 times to obtain T cells.

Cell Culture

4T1 cells (mouse breast cells) were obtained from Institute of Biochemistry and Cell Biology, the Chinese Academy of Sciences (Shanghai, China). The cells were cultured with RPMI 1640 medium with 10% FBS, 1% penicillin-streptomycin, and incubated at 37 °C in a Thermo Scientific cell incubator (Waltham, MA) with 5% CO2.

In Vitro Cytotoxicity

BMDCs were obtained from the hind limbs of C57BL/6 mice according to the previous report. The MTT kit was used to detect the cytotoxicity of different concentrations of Au DENPs-MPC and Au DENPs-MPC/CpG-ODN complexes to BMDCs according to our previous studies. The concentration was ranged from 0-200 μg/mL. The dose of CpG-ODN used for each well was 1 μg. Five parallel experiments were performed in each group.

Cellular Uptake Assays

The BMDCs mixed with Au DENPs-MPC/CpG-ODN complexes (N/P=2) at four different Au DENPs-MPC concentration: 1 μg/mL, 10 μg/mL, 50 μg/mL, or 75 μg/mL. They were seeded into 24 well plates and incubated for 5 h and the BMDCs uptake results (n = 3 for each group) were detected by flow cytometry. CpG-ODN alone under above different concentration was used to as control. After that, the experiment was carried out as described above and the BMDCs uptake results were detected by confocal microscope.

BMDCs Maturation Evaluation

Several different concentrations the Au DENPs-MPC/CpG-ODN complexes (N/P=2) were selected to carry out the BMDCs maturation assay. The Au DENPs-MPC/CpG-ODN complexes were mixed with BMDCs at different concentration (10 μg/mL, 50 μg/mL, or 75 μg/mL) and seeded into 24-well plate. They were incubated for 24 hours. After cultured, the BMDCs were collected and incubated with CD80, CD86 and MHC- II antibodies according to the manufacturer recommended instructions. The expression of various antibodies was tested by flow cytometry and the LPS was used as positive control. The assay was performed 3 times for each sample.

Detection of T Cells Activation

T cells were obtained by nylon wool column method. Mitomycin-C (25 μg/mL) was incubated with mDCs for 45 min, then it was washed away with PBS. Then T cells were cultured in 96-well plate with the above treated BMDCs at the serious ratio of BMDCs: T cells (1: 10, 1: 20, 1: 50, 1: 100, 1:200 separately). The proliferation of T cells was measured by MTT assay. Five parallel samples were set up for each group, and T cells without any BMDCs were used as control. The calculation of stimulus index was carried out by the follow formula: stimulus index (SI) = (A value of the sample to be tested - A value of the culture control group) / (A value of the negative control group - A value of the culture control group).

In vitro antitumor effect of T cells

To imitate the in vivo tumor microenvironment, the activated T cells were co-cultured with 4T1 cells using transwell system which the T cells were added into the upper chamber (5×10^4 cells per mL), and 4T1 cells (5×10^4 cells per mL) were seeded in the lower chamber. The cell viability of 4T1 cells were detected using CCK-8 assay after 24 h co-culture according to previous work. Three parallel experiments were performed in each group.

Statistical Analysis

Data were presented as the means ± standard deviations. One-way ANOVA statistical method was adopted to analyze the experimental results. A p value of 0.05 was selected as the significance level, and the data were marked with (*) for $p < 0.05$, (**) for $p < 0.01$, and (***) for $p < 0.001$, respectively.

Table S1. Physicochemical parameters of the $G_5.NH_2$, $\{(Au^0)_{25}\text{-}G_5.NH_2\text{-}mPEG_{20}\}$ and $\{(Au^0)_{25}\text{-}G_5.NH_2\text{-}MPC_{20}\}$ vectors.

Samples	$G5.NH_2$	$\{(Au^0)_{25}\text{-}G5.NH_2\text{-}m\text{PEG}_{20}\}$	$\{(Au^0)_{25}\text{-}G5.NH_2\text{-}MPC_{20}\}$
Calculated Mw	26010	52094	36830
Mean number of primary amines per dendrimer	98.8	38.6	55.4

Table S2. Zeta potentials and hydrodynamic diameters of Au DENPs-MPC alone and the Au DENPs-MPC/CpG complexes at five different N/P ratios: 1:1, 1:2, 1:4, 1:6 and 1:8.

Samples	ξ-Potential (mV)	Hydrodynamic Size (nm)	Polydispersity Index (PDI)
Au DENPs-MPC	7.59±0.64	176.58±0.68	0.18 ± 0.11
Au DENPs-MPC/CpG-ODN=1	2.28±0.37	218.9±5.45	0.25 ± 0.23
Au DENPs-MPC/CpG-ODN=2	16.89±0.22	229.13±2.33	0.43 ± 0.02
Au DENPs-MPC/CpG-ODN=4	26.67±1.52	231.25±6.81	0.13 ± 0.01
Au DENPs-MPC/CpG-ODN=6	28.98±0.21	314.67±4.7	0.24 ± 0.12
Au DENPs-MPC/CpG-ODN=8	36.15±1.34	351.22±3.64	0.31 ± 0.04

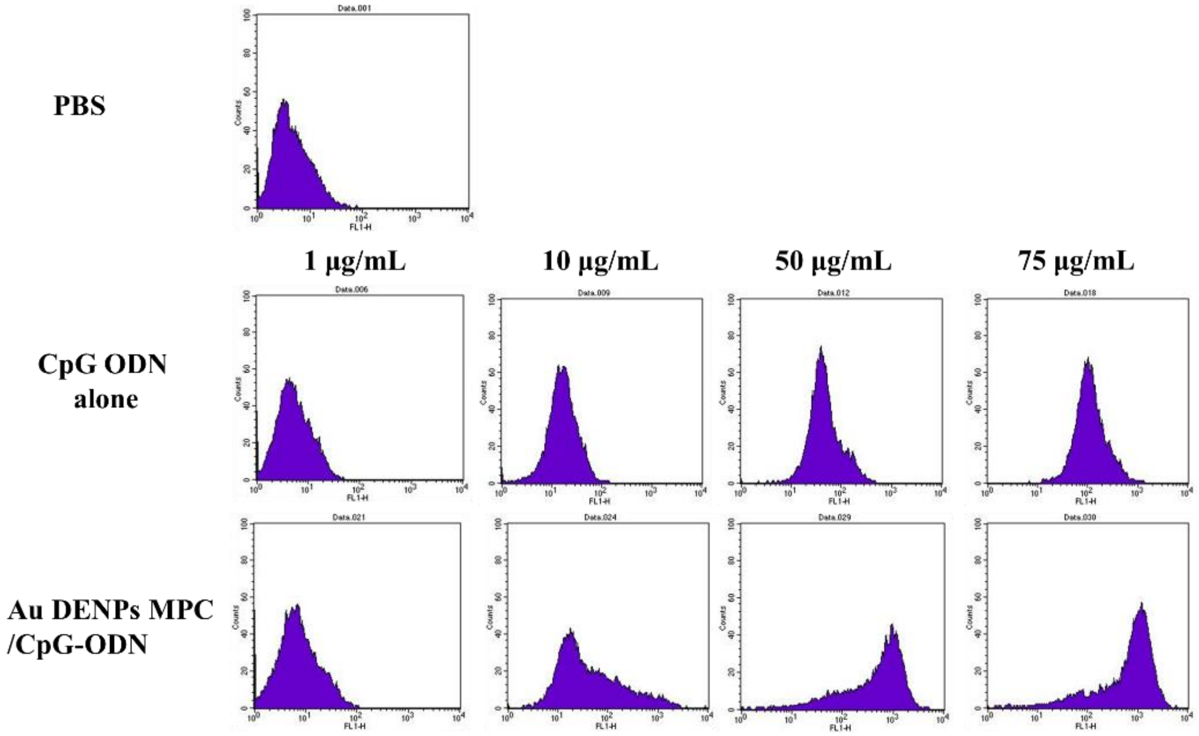

Figure S1. Cellular uptake results of BMDCs detected by flow cytometry. CpG was labeled with FAM (F1 channel).

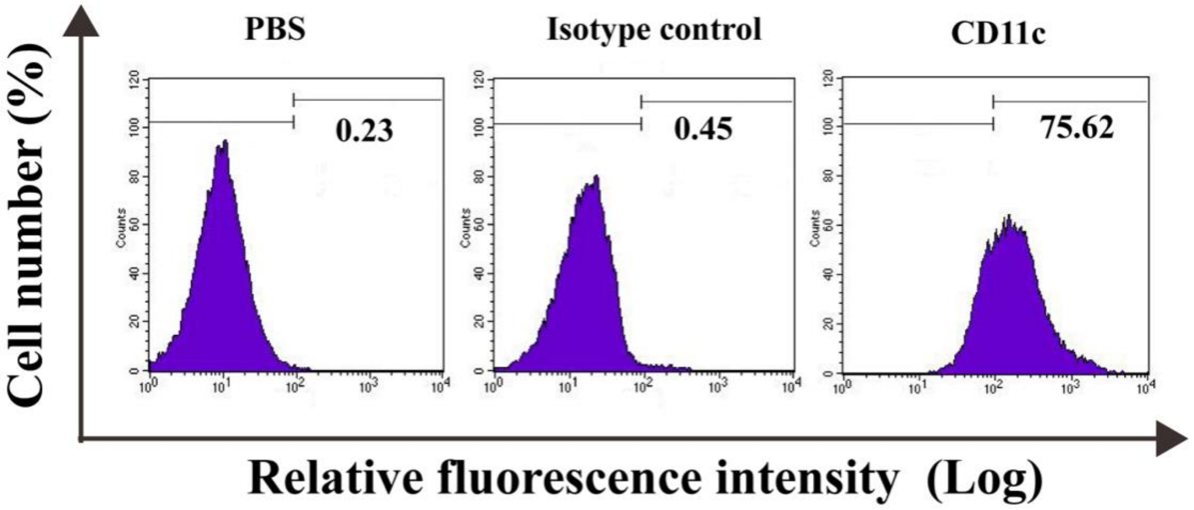

Figure S2. The expression of CD11c on the surfaces of BMDCs related the purity of BMDCs was detected by flow cytometry. CD11c was labeled with FITC (F1 channel).

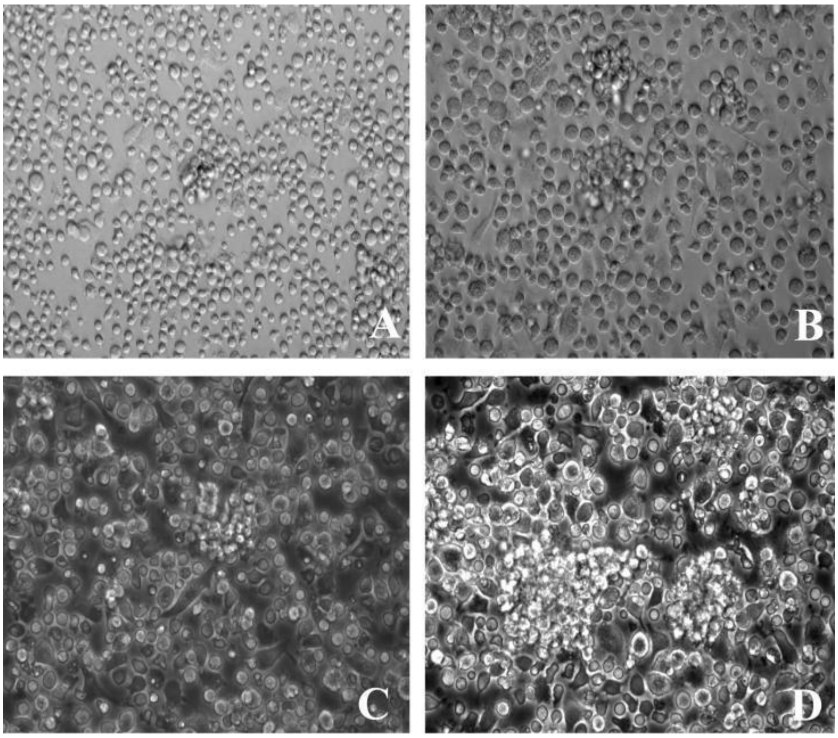

Figure S3. The inverted microscope images of mouse bone marrow-derived dendritic cells after induction for 1 (A), 3 (B), 5 (C), and 7 (D) days.

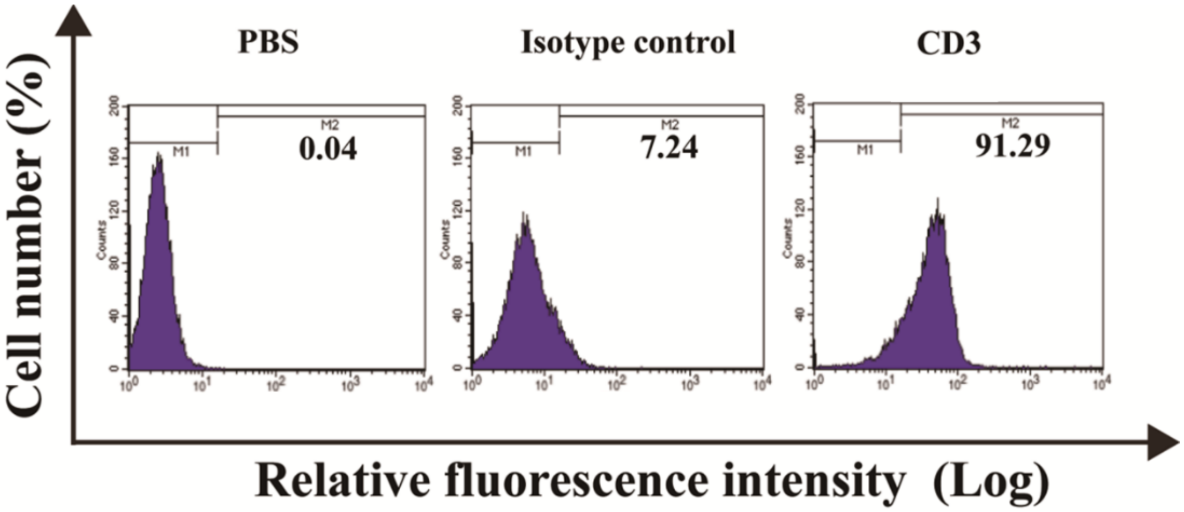

Figure S4. The expression of CD3 on the surfaces of T cells related the purity of T cells was detected by flow cytometry. CD3 was labeled with FITC (F1 channel).

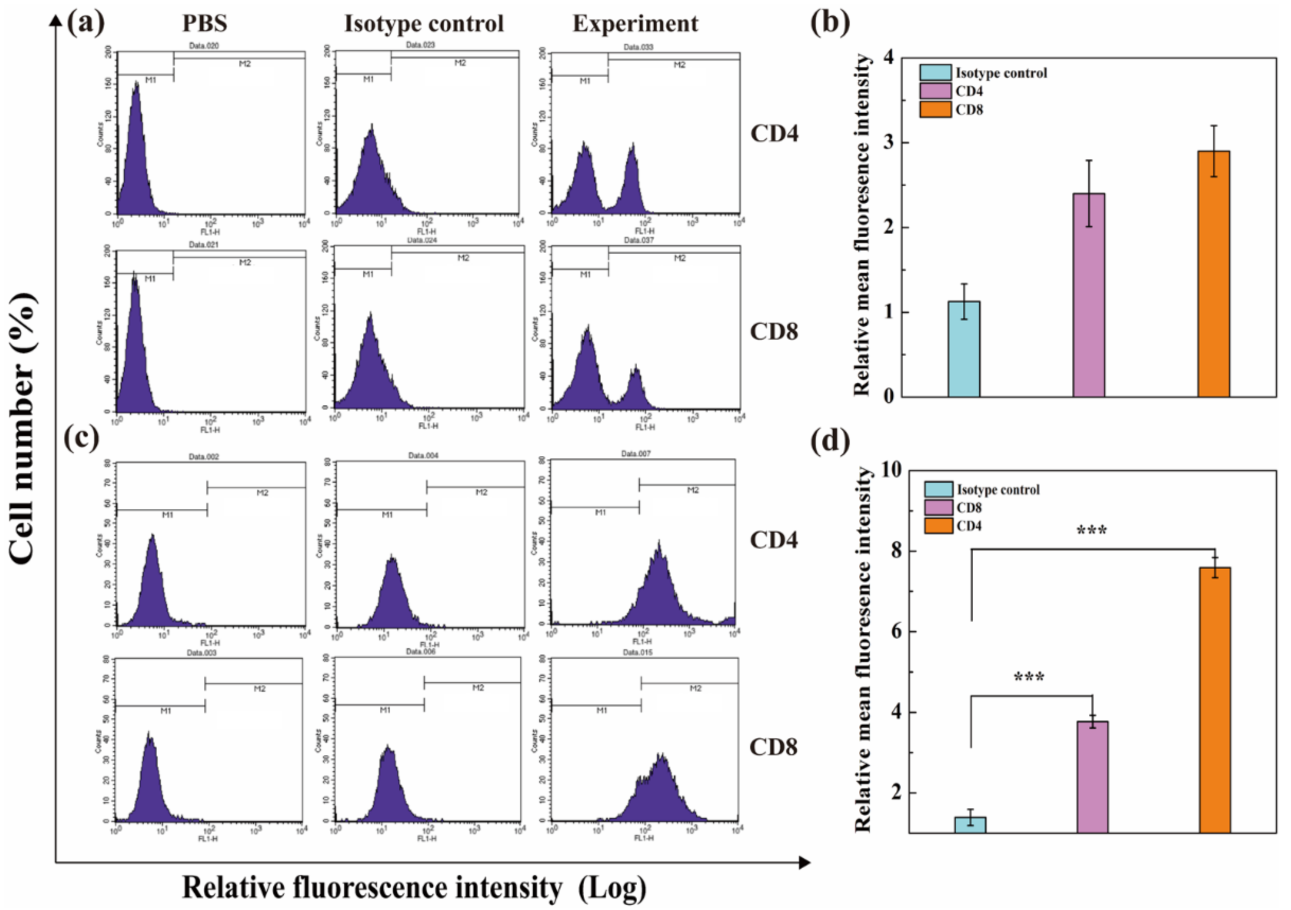

Figure S5. The flow cytometry results of the expression of CD4 and CD8 on the surfaces of T cells. (a) and (b) T cells cultured 3 days without stimulation. (c) and (d) T cells cultured with mBMDCs 3 days. The CD4 and CD8 was labeled with FITC (F1 channel).
